# Supplementary material for: An Antioxidant Supplement Function Exploration: Rescue of Intestinal Structure Injury by Mannan Oligosaccharides after Aeromonas hydrophila Infection in Grass Carp (Ctenopharyngodon idella)
Source: Antioxidants (Basel). 2022 Apr 20;11(5):806. doi: 10.3390/antiox11050806 (PMC9137958; doi:10.3390/antiox11050806)
Supplement: Supplementary file 1 [file antioxidants-11-00806-s001.zip › antioxidants-1672680-supplementary.pdf]

Table S1. Composition and nutrient content of the diet.

Ingredients	%	Nutrient content	%
Fish meal	7.80	Crude protein [4]	28.69
Gelatin	6.00	Crude lipid [4]	5.36
Soybean protein	26.00	n-3 [4]	1.04
Corn starch	19.90	n-6 [4]	0.96
α-starch	24.00	Available phosphorus [4]	0.40
Fish oil	2.34		
Soybean oil	1.81		
Cellulose	5.00		
$Ca (H_2PO4)_2$	1.30		
Vitamin premix [1]	1.00		
Mineral premix [2]	2.00		
MOS premix [3]	1.00		
Choline chloride	1.00		
Ethoxyquin (30%)	0.05		
DL-Met (99%)	0.61		
L-Trp (99%)	0.08		
Thr (98.5%)	0.11		

[1] Per kilogram of vitamin premix (g kg^{-1}): retinyl acetate (500,000 IU g^{-1}), 0.39; cholecalciferol (500,000 IU g^{-1}), 0.40; D, L-α-tocopherol acetate (50%), 23.23; menadione (22.9%), 0.83; cyanocobalamin (1%), 0.94; D-biotin (2%), 0.75; folic acid (95%), 0.42; thiamine nitrate (98%), 0.09; ascorhyl acetate (95%), 9.77; niacin (99%), 4.04; meso-inositol (98%), 19.39; Calcium-D-pantothenate (98%), 3.85; riboflavin (80%), 0.73; pyridoxine hydrochloride (98%), 0.62. All ingredients were diluted with corn starch to 1 kg.

[2] Per kilogram of mineral premix (g kg^{-1}): $MnSO_4 \cdot H_2O$ (31.8% Mn), 2.6590; $MgSO_4 \cdot H_2O$ (15.0% Mg), 200.0000; $FeSO_4 \cdot H_2O$ (30.0% Fe), 12.2500; $ZnSO_4.H_2O$ (34.5% Zn), 8.2460; $CuSO_4 \cdot 5H_2O$ (25.0% Cu), 0.9560; KI (76.9% I), 0.0650; Na_2SeO_3 (44.7% Se), 0.0168. All ingredients were diluted with corn starch to 1 kg.

[3] MOS premix (mg kg^{-1}): premix was added to obtain graded levels of MOS.

[4] Crude protein and crude lipid content were measured value. Available phosphorus, n-3 and n-6 contents calculated according to NRC (2011).

Table S2 Real-time PCR primer sequences [1].

Target gene	Primer sequence (5'→3')	Temperature(°C)	Accession number
Occludin	**Forward:** TATCTGTATCACTACTGCGTCG	59.4	KF193855
	**Reverse:** CATTCACCCAATCCTCCA		
ZO-1	**Forward:** CGGTGTCTTCGTAGTCGG	59.4	KJ000055
	**Reverse:** CAGTTGGTTTGGGTTTCAG		
ZO-2	**Forward:** TACAGCGGGACTCTAAAATGG	60.3	KM112095
	**Reverse:** TCACACGGTCGTTCTCAAAG		
Claudin-b	**Forward:** GAGGGAATCTGGATGAGC	57.0	KF193860
	**Reverse:** ATGGCAATGATGGTGAGA		
Claudin-c	**Forward:** GAGGGAATCTGGATGAGC	59.4	KF193859
	**Reverse:** CTGTTATGAAAGCGGCAC		
Claudin-f	**Forward:** GCTGGAGTTGCCTGTCTTATTC	57.1	KM112097
	**Reverse:** ACCAATCTCCCTCTTTTGTGTC		
Claudin-3c	**Forward:** ATCACTCGGGACTTCTA	57.0	KF193858
	**Reverse:** CAGCAAACCCAATGTAG		
Claudin-7a	**Forward:** ACTTACCAGGGACTGTGGATGT	59.3	KT625604
	**Reverse:** CACTATCATCAAAGCACGGGT		
Claudin-7b	**Forward:** CTAACTGTGGTGGTGATGAC	59.3	KT445866
	**Reverse:** AACAATGCTACAAAGGGCTG		
Claudin-11	**Forward:** TCTCAACTGCTCTGTATCACTGC	62.3	KT445867
	**Reverse:** TTTCTGGTTCACTTCCGAGG		
Claudin-12	**Forward:** CCCTGAAGTGCCCACAA	55.4	KF998571
	**Reverse:** GCGTATGTCACGGGAGAA		
Claudin-15a	**Forward:** TGCTTTATTTCTTGGCTTTC	59.0	KF193857
	**Reverse:** CTCGTACAGGGTTGAGGTG		
Claudin-15b	**Forward:** AGTGTTCTAAGATAGGAGGGGAG	62.3	KT757304
	**Reverse:** AGCCCTTCTCCGATTTCAT		
JAM-A	**Forward:** ACTGTGAGGTGCTTGGAA	61.4	KY780630
	**Reverse:** CTGTTGTGACTGAAGAAGGA		
E-cadherin	**Forward:** GACTGTAACGCTGAAGAGA	61.4	MN661354
	**Reverse:** CTGTGGAGAGGAGATGTTC		
α-catenin	**Forward:** GCAATCTTCTCTCCTTTATCC	61.4	HQ338751
	**Reverse:** ACTTGTGAACTCCAGCAAT		
β-catenin	**Forward:** GTCTGCTTGCCATCTTCA	64.5	MN661349
	**Reverse:** CAGGTTGTGTAGAGTCGTAA		
Nectin	**Forward:** GCCAGTGACCAAGATGAC	61.4	MN661350

	**Reverse:** ACAGTGCCATTCGGATTG		
Afadin	**Forward:** CCTGTGCTCACACTACTG	61.4	MN661352
	**Reverse:** GTCGTTGCCTGGACTATG		
MLCK	**Forward:** GAAGGTCAGGGCATCTCA	53.0	KM279719
	**Reverse:** GGGTCGGGCTTATCTACT		
RhoA	**Forward:** GCAGGACAAGAGGACTATG	63.3	MN661351
	**Reverse:** GTGTTCATCATTCCGTAGGT		
ROCK	**Forward:** AGTCCAAGTCTGCTGCTA	63.3	KY780630
	**Reverse:** CCTCTCCTTCTGCTTCATC		
NMII	**Forward:** AGCCAACTCGTCAATGTC	61.4	MN661353
	**Reverse:** CCTTGGAATACTTCTCTGTCT		
β-actin	**Forward:** GGCTGTGCTGTCCCTGTA	61.4	M25013
	**Reverse:** GGGCATAACCCTCGTAGAT		
GAPDH	**Forward:** GTTACAAGGGAGAAGTTCACCAT	58.0	GQ266395
	**Reverse:** CCGGTAGACTCGACTACATACAG		

[1] *ZO*, zonula occludins; *JAM-A*, junctional adhesion molecule-A; *RhoA*, a small Rho GTPase protein; *ROCK*, the Rho associated protein kinase; *MLCK*, myosin light chain kinase; *NMII*, Non-muscle myosin II.
